# Cost-Effectiveness of Two Government District Hospitals in Sub-Saharan Africa

**DOI:** 10.1007/s00268-017-4007-6

**Published:** 2017-03-27

**Authors:** Caris E. Grimes, Rebekah Law, Anna Dare, Nigel Day, Sophie Reshamwalla, Michael Murowa, Peter M. George, Thaim B. Kamara, Nyengo C. Mkandawire, Andrew J. M. Leather, Christopher B. D. Lavy

**Affiliations:** 1 King’s Centre for Global Health and Health Partnerships, King’s College London and King’s Health Partners, Weston Education Centre, Cutcombe Road, London, SE5 9RJ UK; 2 Medway NHS Foundation Trust, Gillingham, Kent, UK; 3 PGMC, Croydon University Hospital, London Road, London, CR7 7YE UK; 4 Department of Surgery, University of Toronto, Toronto, Canada; 5 London, UK; 6 Thyolo District Hospital, Thyolo, Malawi; 7 Bo District Hospital, Bo, Sierra Leone; 8 Connaught Hospital, Freetown, Sierra Leone; 9 Department of Surgery, College of Medicine and Allied Health Sciences, Freetown, Sierra Leone; 10 College of Medicine, University of Malawi, Mahatma Gandhi Road, Blantyre, Malawi; 11 School of Medicine, Flinders University, Adelaide, Australia; 12 King’s Centre for Global Health and Health Partnerships, King’s College London and King’s Health Partners, London, UK; 13 King’s College London, London, UK; 14 University of Oxford, Oxford, UK

## Abstract

**Background:**

District hospitals in sub-Saharan Africa are in need of investment if countries are going to progress towards universal health coverage, and meet the sustainable development goals and the Lancet Commission on Global Surgery time-bound targets for 2030. Previous studies have suggested that government hospitals are likely to be highly cost-effective and therefore worthy of investment.

**Methods:**

A retrospective analysis of the inpatient logbooks for two government district hospitals in two sub-Saharan African hospitals was performed. Data were extracted and DALYs were calculated based on the diagnosis and procedures undertaken. Estimated costs were obtained based on the patient receiving ideal treatment for their condition rather than actual treatment received.

**Results:**

Total cost per DALY averted was 26 (range 17–66) for Thyolo District Hospital in Malawi and 363 (range 187–881) for Bo District Hospital in Sierra Leone.

**Conclusion:**

This is the first published paper to support the hypothesis that government district hospitals are very cost-effective. The results are within the same range of the US$32.78–223 per DALY averted published for non-governmental hospitals.

## Introduction

We have previously argued for investment in district hospitals in low-income countries, as key to the development of sustainable, long-term healthcare solutions [1]. District hospitals need investment as part of health system strengthening and the need to progress towards universal health coverage (UHC) [2] as well as to achieve the sustainable development goals (SDGs) [3].

District hospitals are also key to strengthening surgical care, or universal coverage of essential surgery (UCES) [4]. Provision of essential surgical procedures, largely at district hospitals, could avert an estimated 1.5 million deaths per year, or 6–7% of all avertable deaths in low- and middle-income countries (LMICs) [4]. The Lancet Commission on Global Surgery (LCoGS) highlighted the huge burden of untreated conditions requiring surgical care, with mortality rates from lack of surgical access significantly exceeding mortality rates from key infectious diseases [5]. Surgery is required by all areas of medicine—a proportion of patients in every Global Burden of Disease (GBD) category required a procedure in an operating room [6]. One LCoGS target is that 80% of the global population should have access within 2 h to a facility that can perform the Bellwether Procedures (emergency caesarean section, laparotomy or open fracture fixation). This ambitious target will require significant investment in district hospital care. Anecdotal evidence suggests that improvement in surgical district hospital care would have a general effect on health by acting as an “enabler,” to raise the overall quality of health care and encouraging patients to seek care for non-surgical conditions [7].

However, district hospital provision is in crisis, particularly in Africa, both in terms of the number needed per population [2], and the quality and presence of key basic medical and surgical facilities [8–10]. If we are to meet global health targets, as well as the global surgical targets, there will be a need for massive investment in district hospitals, both in the number of them and their ability to provide quality health care.

Cost-effectiveness studies to date have not assessed the cost-effectiveness of government district hospitals although they are thought to probably be highly cost-effective [4]. To date, four studies have been performed, most from the perspective of the district hospital as a surgical platform [11–14], but all of which were in non-governmental sector hospitals, and three of the studies included hospitals that did not provide obstetric care. Overall, the cost-effectiveness for non-governmental trauma hospitals was US$32.78–223 per disability-adjusted life year (DALY) averted. The exclusion of obstetric care may be crucial as obstetric care was shown to be the source of the highest number of averted DALYs in the McCord study in rural Bangladesh [11]. Debas et al. [15] estimated that the ideal first-level referral hospital as a platform for surgical care provision should be highly cost-effective at an estimated US$33 per DALY averted for sub-Saharan Africa. Our aim was to determine the cost-effectiveness of an entire government district hospital in sub-Saharan Africa.

## Methods

### Setting and data collation

We chose two busy rural government district hospitals in two different countries of sub-Saharan Africa—Sierra Leone and Malawi. The hospitals were chosen because they were felt to maintain good records and were similar in size and capacity. The characteristics of these two hospitals are shown in Table 1. We estimate that we captured data from at least 90% of all admissions.

**Table 1:** Hospital characteristics 2012

	Thyolo DH, Malawi	Bo DH, Sierra Leone
Population catchment	600,000 approx	600,000 approx
Number of beds	350	450
Staffing		
Doctors	3	1
Medical officers	0	4
Clinical officers	31	n/a
Nursing staff	116	229
Allied medical staff	40	94
Administration staff	20	10
Other (cleaners, drivers, etc.)	300	49

### Thyolo District Hospital, Malawi

Malawi is a landlocked country in East Africa and has a population of approximately 15 million of which 85% live in rural areas. Thyolo District Hospital in southern Malawi is a 350-bed government district hospital catering for a population of approximately 600,000. It has one fully qualified doctor, the District Medical Officer, who has the responsibility for the overall running of the hospital with the majority of the clinical work being performed by 20 paramedics (clinical officers) supported by nursing staff. It has two operating theatres, but only one that was in regular use during the period covered by this study. In Malawi, medical and surgical treatment is free at the point of delivery and patients therefore do not have to pay for equipment, patient stay, or any costs associated with their care including the operative procedure.

### Bo District Hospital, Sierra Leone

Sierra Leone in West Africa has a population of approximately 6 million with 60% of the population living in rural areas. Bo District Hospital in Sierra Leone is a 450-bed government district hospital catering for a population of approximately 600,000. It has two operating theatres, but at the time of data collection, only one was in use.

In Sierra Leone, although health care is free at the point of delivery for under 5’s, pregnant and lactating women, all other patients have to pay for costs related to bed stay, equipment, supplies, medications, investigations and the operation. The costs of the operation are divided into minor, intermediate and major procedures and are set centrally by the government each year.

### Data collection

Whole hospital inpatient data were collected retrospectively from two separate 3-month periods, representing rainy and dry seasons, through rigorous review and analysis of logbooks from all wards and theatres. The different sets of data were cross-referenced and duplicates removed.

In Sierra Leone, all hospital data were collected for the months of July to September 2012 inclusive and February to April 2013 inclusive to capture both rainy and dry seasons. In Malawi, all hospital data were collected for the months of January to March 2013 and April to July 2013.

### DALY calculation

For each diagnosis, we used a weighting system used by previous authors [11–13, 16, 17] to determine the likely threat to life without treatment, the likelihood of permanent disability and the likely efficacy of treatment (Table 2). Weights for each operation were estimated using a Delphi method of local doctors and experts in each medical discipline. Disability-adjusted life years averted were calculated using the following formula. For deaths averted, we calculated

**Table 2:** Weightings

Weighting given	Risk of death or permanent disability	Treatment efficacy
0	Condition fatal or permanently disabling <5% of the time	<5% chance of permanent cure
0.3	Condition fatal or permanently disabling 5–50% of the time	5–50% chance of permanent cure
0.7	Condition fatal or permanently disabling 50–95% of the time	50–95% chance of permanent cure
1	Condition fatal or permanently disabling >95% of the time	>95% chance of permanent cure

$${\text{Life}}\;{\text{expectancy}} \times {\text{likely}}\;{\text{threat}}\;{\text{to}}\;{\text{life}} \times {\text{efficacy}}\;{\text{of}}\;{\text{treatment}}$$

This formula is the same as has been used to calculate life years saved in other papers [18]. For those conditions that predominantly cause disability, we used the formula

$${\text{Life}}\;{\text{expectancy}} \times {\text{risk}}\;{\text{of}}\;{\text{permanent}}\;{\text{disability}} \times {\text{disability}}\;{\text{weight}} \times {\text{efficacy}}\;{\text{of}}\;{\text{treatment}}$$

We used local life expectancies from the WHO Tables and disability weights from the Global Burden of Disease study [19]. We did not use age weighting or discounting in the base case analysis in line with the GBD study [20].

### Calculation of costs

#### Capital costs

Actual building costs for Thyolo, and estimated building costs for Bo were obtained and depreciated over 20 years using a straight-line method. Equipment costs were estimated at 15% of the building costs and depreciated over 7.5 years [21].

#### Recurrent costs

For Thyolo, actual salaries were obtained from the hospital for all medical and non-medical staff within the hospital for the financial year beginning 2012. Other recurrent costs, including fuel, utilities and maintenance were obtained from the hospital on an “ideal” level, i.e. what was requested from the Ministry of Health rather than what was obtained. Equivalent estimates for Bo were obtained from reports on health system financing in Sierra Leone [22, 23].

#### Costs of medicines

Government district hospitals in sub-Saharan Africa tend to be underfunded, and therefore, we calculated “ideal” treatment rather than “actual” treatment. For each diagnosis, we identified the best treatment according to international or published guidelines. We costed the medicines using the WHO International Drug Price Indicator guide [24, 25], and calculated the costs of other materials, e.g. catheters, cannulas and intravenous lines from local price lists. As far as possible, we costed for the full course of treatment for all acute conditions, even if part or most of this would usually be delivered on an outpatient basis, e.g. 6 months of triple therapy for uncomplicated pulmonary tuberculosis, because we argue that partial treatment of such conditions could be detrimental. For chronic, long-term conditions such as epilepsy, we costed for inpatient or intravenous treatment and then for two weeks of outpatient oral daily medication, accepting that lifelong treatment for disease control would need to be found for each patient but was not part of inpatient hospital costs and therefore not part of our study. We assumed that most infections (e.g. pneumonia) would require minimum three days of intravenous high-dose antibiotics, followed by completion of an oral course, except for severe infections such as meningitis, where we costed for a minimum of a one week course of intravenous high-dose antibiotics. We used a mean cost per major surgical procedure of US$179 (2012 US$), used by Verguet et al. [26] and based on the unit cost of a caesarean section [27] as has been used in previous studies. All costs were converted to international dollars using the purchasing power parity for Sierra Leone and Malawi in 2012 [28].

### Sensitivity analysis

We performed sensitivity analysis by recalculating the DALYs averted using the World Health Organization Guidelines for Cost-Effectiveness Analysis [29] with GBD disability weights and with and without age weighting and discounting at 3%, and using ideal life expectancies from the GBD study [30].

### Ethical approval

This study received research ethics committee approval from the College of Medicine Research Ethics Committee, Malawi, and the Sierra Leone Ethics Committee.

## Results

Table 3 outlines the estimated total annual costs for each hospital. A total of 97,844 DALYs were averted by Thyolo District Hospital compared with 36487 in Bo District Hospital (Table 4). The highest number of averted DALYs were in paediatrics (42 and 59%). 35% of all DALYs were averted in Bo by surgery and obstetrics/gynaecology, and 24% of DALYs in Thyolo. Total cost (US$)/DALY averted was 26 (range 17–66) for Thyolo and 363 (range 187–881) for Bo (Tables 5, 6).

**Table 3:** Annual costs for 2012 in US$

Item	Thyolo DH, Malawi	Bo DH, Sierra Leone
Capital costs		
Buildings	632,152	250,000
Equipment	270,923	107,142
Salaries	410,179	7,743,956
Recurrent costs (utilities, fuel, admin, maintenance, etc.)	1,836,591	5,416,362
Materials and medicines	226,272	160,719
Total (US$) per year	3,376,117	13,678,178

**Table 4:** DALYs averted per specialty in 6 months

Specialty	Number of DALYs averted (%)	
	Thyolo	Bo
Medicine	32,780 (34)	2301 (6)
Obstetrics/gynaecology	20,577 (21)	7138 (20)
Paediatrics	40,592 (42)	21,669 (59)
Psychiatry	534 (1)	21 (0)
Surgery	3361 (3)	5073 (14)
Paediatric surgery	n/a	285 (1)
Total	97,844	36,487

**Table 5:** Sensitivity analysis

Method	DALYs averted Malawi^a^	DALYs averted Sierra Leone^a^
Baseline	97,843.55	36,487.36
WHO methodology—no age weighting/discounting	43,563.63	14,713.39
WHO methodology—with age weighting and discounting	25,682.63	7759.10
Baseline using local life expectancies	64,435.34	18,835.8

^a^Over 6 month period of study

**Table 6:** Overall cost-effectiveness per annum

Method	Cost/DALY averted Malawi	Cost/DALY averted Sierra Leone
Baseline (local life expectancies)	26	363
WHO methodology—no age weighting/discounting	39	465
WHO methodology—with age weighting and discounting	66	881
Baseline using ideal life expectancies	17	187

## Discussion

This study suggests that government district hospitals in sub-Saharan Africa may be very cost-effective as defined by the World Health Organization [31] and in line with previous estimates. However, there are a number of limitations and discrepancies with our study. Our data do not show as high a proportion of DALYs averted from obstetric or surgical care than previously reported and there are likely to be a number of reasons for this. From the provider perspective, the provision of safe surgical care requires the presence of a number of factors, including trained surgical and anaesthetic providers, infrastructure, equipment and supplies for surgery and anaesthesia, de-contamination and sterilisation facilities, a safe and affordable blood supply, and trained nursing staff. From the patient perspective, patients present late with surgical conditions in Africa and are therefore not always amenable to surgical treatment or correction. Many patients never present at all for a variety of reasons ranging from the quality of the roads, medical beliefs and direct and indirect costs of treatment [32]. It is likely that fully funded, fully equipped hospitals would attract significantly more patients with conditions requiring surgical attention. In addition, we noted in a previous study the high proportion of patients with surgical conditions who abscond from hospital after diagnosis—38% with bowel obstruction, 39% with peritonitis and 20% with ectopic pregnancy [33] with previous studies on absconding suggesting there may be both cultural and financial reasons for this [34, 35]. Finally, comparison with the previous studies is difficult because of the widely differing contexts—both in the caseload of the hospital and the local disease demographics in different countries.

Further limitations to this study occur with respect to calculating DALYs and costs. Although both hospitals were highly cost-effective, the overall cost/DALY averted was quite different when the two are compared. There are a number of possible reasons for this. Surgical services were also available in nearby non-governmental hospitals, potentially reducing uptake for certain surgical conditions in government hospital services. As discussed in our previous paper, a nearby non-governmental hospital in Bo providing maternal health services meant that a significant number of obstetric admissions were referred there rather than treated in the government hospital. Conversely, national health policy in Sierra Leone provides free health care for under 5’s and pregnant/lactating women, which is likely to have driven up the proportion of DALYs averted in Bo for these groups. However, although these were high (59 and 20% of all DALYs averted), they percentages were similar in Thyolo (42 and 21%). Costs are also problematic to calculate in this context as many low-income countries have very underfunded district hospitals, and therefore, calculating accurate costs for services is challenging. Furthermore, some NGOs provide additional funding either for salaries or for specific diseases. The much higher cost/DALY averted for Bo District Hospital was due to higher estimated salary costs and recurrent costs which may in part have been because we used estimated costs for these as it was not possible to obtain either actual or ideal costs.

DALYs themselves are problematic as discussed in detail elsewhere. We used estimates of likely threat to life and risk of permanent disability, along with likely effectiveness of treatment, based on Delphi method of local doctors and international experts. However, these may not have reflected the actual outcomes for individual patients. We attempted to calculate ideal treatment for a patient based on the diagnosis, and using the WHO Price Indicator, but this does not necessarily reflect the actual costs at local level of providing such treatment. Furthermore, often individual patients presenting with the same condition may require treatment for different lengths of time, or may develop complications of an illness which may require different treatment. We used a mean cost of a major surgical procedure of US$179, based on previous studies, but major surgical procedures can vary widely in actual costs, and the same procedure may cost differently in different patients. Certainly the study would have been methodologically stronger if it had been possible to perform it as a prospective study, observing actual costs to both provider and patient, as well as actual outcomes, but this was not practical with the resources available.

Finally, large numbers of patients were seen in both hospitals in the outpatient department—over a thousand a month in Thyolo alone—and it was not possible to incorporate these into the study. Thus, the overall number of DALYs averted if the outpatient workload as well as the inpatient workload had been taken into account would have probably made the overall cost-effectiveness higher.

## Conclusion

This is the first published evidence of the cost-effectiveness of government district hospitals in sub-Saharan Africa and adds weight to the argument that Ministries of Health and the Donor Community should invest in government district hospitals in order to reduce mortality and disability from surgical conditions.
